# Explorative Data Analysis of *Drosophila suzukii* Trap Catches from a Seven-Year Monitoring Program in Southwest Germany

**DOI:** 10.3390/insects9040125

**Published:** 2018-09-24

**Authors:** Felix Briem, Anto Raja Dominic, Burkhard Golla, Christoph Hoffmann, Camilla Englert, Annette Herz, Heidrun Vogt

**Affiliations:** 1 Julius Kühn-Institut (JKI), Federal Research Centre for Cultivated Plants, Institute for Plant Protection in Fruit Crops and Viticulture, Schwabenheimer Straße 101, 69221 Dossenheim, Germany; heidrun.vogt@julius-kuehn.de; 2 Julius Kühn-Institut (JKI), Federal Research Centre for Cultivated Plants, Institute for Strategies and Technology Assessment, Stahnsdorfer Damm 81, 14532 Kleinmachnow, Germany; anto.raja@julius-kuehn.de (A.R.D.); burkhard.golla@julius-kuehn.de (B.G.); 3 Julius Kühn-Institut (JKI), Federal Research Centre for Cultivated Plants, Institute for Plant Protection in Fruit Crops and Viticulture, Geilweilerhof, 76833 Siebeldingen, Germany; christoph.hoffmann@julius-kuehn.de; 4 Julius Kühn-Institut (JKI), Federal Research Centre for Cultivated Plants, Institute for Biological Control, Entomology and Beneficial Insects, Heinrichstraße 243, 64287 Darmstadt, Germany; camilla.englert@julius-kuehn.de (C.E.); annette.herz@julius-kuehn.de (A.H.)

**Keywords:** Drosophilidae, host plants, insect behavior, insect traps, invasive species, online DB, population dynamics

## Abstract

Over the last decade, *Drosophila suzukii* Matsumura, an invasive pest of soft-skinned fruits, gradually established itself in Europe, often resulting in significant economic losses. In 2011, when *D. suzukii* was first described for Germany, the Julius Kühn Institut (JKI) started a monitoring program in southwest Germany to study the occurrence and activity of the fly. Capture data from late 2011–early 2018 from 100 traps were analyzed for the effect of weather and immediate habitat on trap captures at different times of the year. We identified five phases in the annual population development cycle of *D. suzukii*. We found that the mild winter of 2013/2014 helped the thorough establishment of *D. suzukii* in Germany. Habitat types in the immediate vicinity of the trap and local weather conditions had a strong influence on trap captures. Forest borders and hedges were found to provide adequate overwintering shelter for the flies. Trap captures in forests and hedges were generally higher than those of vineyards and orchards, even during the fruiting seasons. Summer capture rates were correlated with the number of heat days and precipitation. We also discuss briefly the limitations of using trap captures as representative of fly density in the field.

## 1. Introduction

*Drosophila suzukii* Matsumura 1931 (Diptera: Drosophilidae), commonly called spotted wing *Drosophila*, is an invasive insect pest of soft-skinned fruits native to Southeast Asia. As a highly polyphagous pest species, adults are attracted to a wider range of odors of ripening and ripe fruits than most drosophilids [1]. In contrast to most Drosophilidae, female *D. suzukii* possess a saw-like sclerotized ovipositor, enabling oviposition into healthy, ripening and ripened fruits [2,3], resulting in heavy economic losses to fruit growers [4,5]. Many economically important soft and stone fruits (e.g., raspberries and blackberries: *Rubus* spp., strawberries: *Fragaria ananassa*, cherries: *Prunus avium*, *P. cerasus*, plums: *P. domestica*, elderberry: *Sambucus* spp.), some susceptible grapevine varieties, as well as numerous wild or ornamental fruits (e.g., *P. serotina*, *Viscum album*, *Rubus* spp., *Basella alba*, [6,7,8,9,10,11] are known hosts. While a high number of reproduction hosts is known from fruit sampling and lab studies, the complete feeding ecology is yet to be described. Early attempts to identify the ingested plant DNA and microbes in the guts of adult individuals have been successful [12,13]. Further studies using these techniques will help to understand the whole feeding ecology of this pest species. 

Continental USA (California) and Southern Europe (Italy and Spain) recorded *D. suzukii* in 2008 for the first time [2,14,15]. It spread rapidly the following years, with periodic reports of economic damage in cultivated soft and stone fruits around the world [4,5,6].

In 2011, the Julius Kühn-Institut (JKI) established a monitoring program in southwest Germany in the vicinity of the JKI in Dossenheim, Siebeldingen and Darmstadt expecting the arrival of *D. suzukii*. Monitoring traps were randomly installed in different fruit crops, forested areas and hedges, in experimental fields and in the direct vicinity of the institutes. First recorded observations of *D. suzukii* in Germany date from late summer 2011 from private observations in Bavaria and from the JKI monitored traps [16,17].

In spring 2016, the JKI launched the online platform DrosoMon (http://drosomon.julius-kuehn.de/), which serves as a database and visualization platform for monitoring the occurrence and activity of *D. suzukii*. Currently, several other institutes and plant protection services from all over Germany, as well as institutions from other European countries have joined this project. DrosoMon helps with the harmonization of the datasets and simplifies the comparability of trap data between monitoring systems. DrosoMon started with the data of the three JKI sites located in the northern part of the upper Rhine valley, an important fruit- and wine-growing region. Many of the fruit crops are susceptible to *D. suzukii* infestation, and the resulting damages lead to substantial economic losses. This region is characterized by small-scale agriculture thriving under the humid Atlantic climate with warm summers and moderate winters [18,19] and provides diverse habitats and retreats for *D. suzukii*. The climate differs significantly from that of other fruit- and wine-growing regions like the Pacific Northwest with hot dry summers and cold winters or the coastal climates of North America with their mild winters [20,21,22,23,24,25]. However, it is quite similar to the moderate summer and winter climate of Northern Italy, where *D. suzukii*’s presence since 2009 has been related to episodic damages to fruit harvests [25,26,27,28].

When *D. suzukii* was first recorded in Europe, it was suggested that cold winters and hot dry summers are unsuitable for this species [15,21]. Consequently, it was supposed that only low numbers of individuals might overwinter at sheltered sites [3,6]. Previously published studies already showed that *D. suzukii* is well adapted to various weather conditions [29] and defined weather indices for their development, mortality and population dynamics [20,27,30,31]. Seasonal dimorphism of winter and summer morphs has been put forth as an adaptation to survive hot and dry summers and overwinter colder winters [6,32].

In the present study, a number of the above-mentioned weather indices were evaluated for their impacts on *D. suzukii* trap captures in the upper Rhine Valley. We describe for the first time the seasonal trap-activity of *D. suzukii* in various habitats of the highly diverse and fragmented upper Rhine valley.

The objectives of this study were (i) to study the spatio-temporal trap activity of *D. suzukii* in cropped and wild habitats, (ii) the relationship between land use and the seasonal and annual capture rates and (iii) the impact of weather on capture rates.

## 2. Materials and Methods

### 2.1. Study Area: Topography and Weather

**Figure 1:**
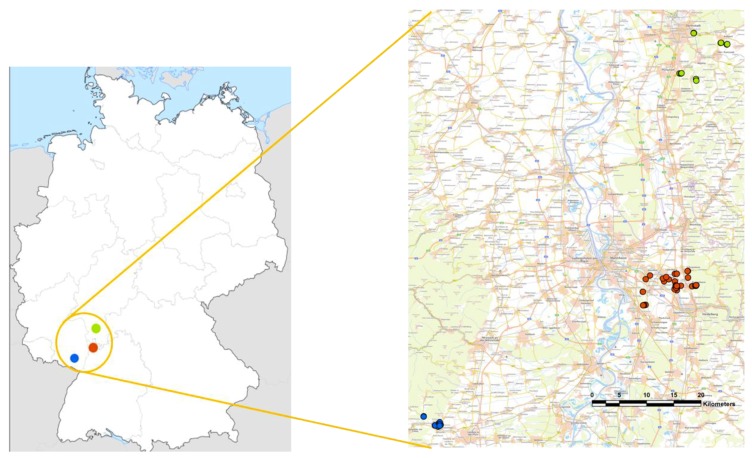
Map of Germany (left) showing the monitoring area (orange inset) and the location of the monitoring traps belonging to each of the three Julius Kühn-Institut (JKI) work groups (red = JKI Dossenheim (Dos), blue = JKI Siebeldingen (Sie) and green = JKI Darmstadt (Dar)). Source: © GeoBasis-DE/BKG 2018.

The monitored areas were clustered around the “Institute for Plant Protection in Fruit Crops and Viticulture” with its sites in Dossenheim (JKI Dos) and Siebeldingen (JKI Sie) and the “Institute for Biological Control” in Darmstadt (JKI Dar), covering an area of ~75 km^2^, in southwest Germany (Figure 1). This region is characterized by the central Rhine rift valley with the Palatinate Forest to the west and the Forest of Odes to the east. About half of the agricultural area in the upper Rhine valley is dedicated to specialized crops (e.g., asparagus, blackberries, cherries, corn, plums, strawberries, raspberries, hops, tobacco, various vegetables and wine). It is also home to the three largest wine-growing regions in Germany: Baden, the Palatinate and Rhine-Hesse.

The upper Rhine falls under the ‘humid, warm temperate’ climate (Cfb) of the Köppen and Geiger climate classification: oceanic with warm summers and mild winters, with rainfall distributed throughout the year [18,19]. During the monitoring period (2011–2018), the mean annual temperature was 11 (±7.3) °C, and the mean annual precipitation was 746 (±103) mm. This period also recorded some of the hottest summers, with 2015 being the hottest ever recorded in Germany (German Weather Service, DWD).

### 2.2. Trap Designs and Placement

**Figure 2:**
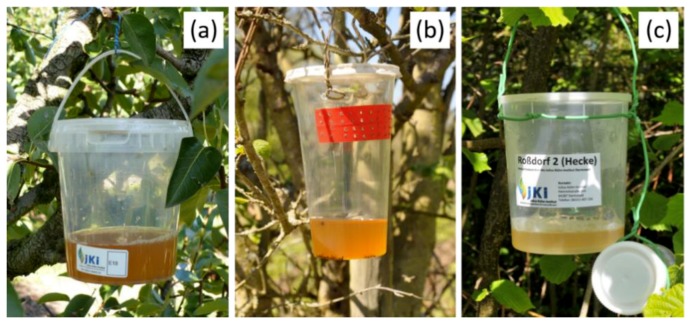
Monitoring traps: (**a**) JKI Dos, (**b**) JKI Sie, (**c**) JKI Dar. Photos: F.B., C.H., C.E.

The traps of the three sites (Figure 2) were quite similar in design and bait used, following the guidelines from the JKI’s *D. suzukii*-website (https://drosophila.julius-kuehn.de) with minor variations in the size and shape of the cups. The JKI Dos trap [33] (Figure 2 and Figure S1) was a clear plastic cup (JETB 850, Jokey Plastik Wipperfürth GmbH, Wipperfürth, Germany) with an airtight lid. A stencil (Figure S1b) was used to drill holes uniformly (21 holes in two rows, Ø = 2.5 mm, distance between the holes = 20 mm) into the upper third of the cup, leaving 1/3 of the cup circumference unperforated to allow complete decanting of the trap contents. The traps were filled with 200 mL of unfiltered apple cider vinegar (ACV) (acetic acid content 5%, K-Classic, Kaufland, Neckarsulm, Germany), diluted to 40% with tap water. Finally, a drop of odorless detergent (1L = 0.025%) was added to the mixture to reduce surface tension. When temperatures were close to freezing, 5% NaCl was added to the trap mixture to avoid the freezing of the bait liquid. Bioassay tests showed no statistical differences between 0% and 5% NaCl in the attractiveness of the mixture, whereas differences were significant between 0% and 10% NaCl for both females (W = 133, *p*-value < 0.001) and males (W = 133, *p*-value < 0.001). The JKI Sie trap (Figure 2) (Econo Plastic cup 500 mL, Huthamaki, Alf, Mosel, Germany) had 33 holes (Ø = 2.0 mm, distance between the holes = 5 mm) burned with a soldering iron into the upper third of the cup, leaving 3/4 of the cup circumference unperforated. The perforated area was reinforced with red tape before burning holes. The traps were filled with the same ACV mixture (200 mL) as the JKI Dos trap. The JKI Dar trap (Figure 2) was an air-tight, clear plastic cup (polypropylene, 1000 mL, H. Hermann Rotert GmbH and Co. KG, Bad Iburg, Germany) with 10 holes (Ø = 2.5 mm, distance between the holes = approximately 28 mm) drilled on the side in the same fashion as the JKI Dos trap. The baiting liquid was unfiltered ACV (acetic acid content 5%, Alnatura, Bickenbach, Germany) diluted to 40% with tap water and a drop of detergent (Elina, Karlsbach, Germany). The traps were filled with 100 mL of the mixture.

Trap sites were chosen randomly to represent a wide range of host plants and/or potential overwintering grounds in the immediate vicinity. The traps were installed in semi-natural habitats, mainly hedges with wild host plants of *D. suzukii*, forested, agricultural and urban areas, in the vineyards of JKI Sie and in the experimental orchards of JKI Dos and JKI Dar.

The JKI Dos traps where changed fortnightly, JKI Sie traps weekly during summer, spring and autumn and fortnightly in December and January. JKI Dar traps were changed weekly between March and September and fortnightly during the rest of the year. This schedule was followed as much as possible with minor variations depending on weather conditions and trap accessibility.

### 2.3. Species Identification

The trap content was decanted onto a sieve (mesh size <0.5 mm) to filter out the flies from the liquid. Larger insects were manually separated and the rest transferred into petri dishes where they were identified using an identification key [34] and counted under a stereo microscope (Stemi 2000, Zeiss, Oberkochem, Germany). When the catch was >5 mL with over ~80% estimated *D. suzukii*, the contents were transferred to petri dishes of a diameter of 6 cm or 14 cm depending on the volume of the catch (less than or greater than 15 mL, respectively). The smaller petri dish was split into 6 equal segments and the bigger one into 8 with metal wires attached to a plastic ring insert (Figure S2). *D. suzukii* counting was carried out for two randomly selected diametrically opposed sections and then extrapolated to the whole petri dish. At JKI Sie and JKI Dar, every single fly was checked and counted.

### 2.4. Monitoring Data from DrosoMon

The following trap-specific characteristics were entered in the online database DrosoMon: coordinates of the trap location, trap design, bait used, plant on which the trap is hung and the habitat type in the direct vicinity of the trap. Traps were placed on 56 different host plants, located in six different habitat types (Table 1) [35]. Capture data for each trap deployment were entered as start and stop dates and the number of male and female *D. suzukii* captured. Based on the capture rates in different periods of the year and the current knowledge on the phenology of *D. suzukii* [6,20,22,36], the captures were categorized into five phenology/seasonal classes (Table 2) representing either their activity patterns or the annual population phases of the fly. Thus, a typical *D. suzukii*-year in this region starts on the 12 March of each year (Table 2). Though each institute set a fixed trap deployment period, they varied over the monitoring period and between institutes. The median collection period for the trap contents for JKI Dos, JKI Sie and JKI Dar was 14 (range: 4–28), 7 (range: 4–44) and 7 (range: 5–48) days, respectively.

### 2.5. Exploratory Statistical Analysis

Fly captures were standardized by calculating the sum of females and males captured per day per trap for each trap deployment. In all subsequent analysis, the sum of individuals/day/trap has been used unless indicated. The daily captures per trap were summarized by sites, host plants and sampled habitat type over years and seasons independently and visually explored for patterns and variations. Annual population dynamics were described by seasons and sampled habitat types. Based on the typical capture patterns over the year, we consider the period from March 12–March 11 of the following year as one annual population cycle, referred to as *D. suzukii*-year hereafter. Traps were sorted in order of their highest capture rates, and the top five traps were identified and manually checked for location effects by way of surrounding habitats/land use types to explain their disproportionately high captures. The numbers of females and males were converted to percentages for comparison. Seasonal sex ratios were compared and tested for significant differences using negative binomial regression from the R: MASS package [37].

Daily gridded weather data from the DWD was mapped to the location of the traps, and a number of weather indices shown to affect *D. suzukii* growth and development [20,27,30,31,32,38,39] were calculated for each trap deployment (Table 3). The capture rates were regressed against the weather indices using negative binomial regression to account for the extreme overdispersion in the dataset. Hot days and optimal development days were regressed against late spring and summer captures. Frost, ice and winter mortality days were regressed against early spring, autumn and winter captures. The mean of daily mean temperature was regressed against captures from all seasons. All analyses were performed in R 3.3.3 (R Development Core Team 2016), and graphs were done using the ggplot2 package [40].

## 3. Results

The number of traps monitored varied across the years from 25 in 2011 to 93 in 2016–2017 (Table 4). The traps were exposed for a total of 116,602 days (min = 4, max = 48) and captured 756,768 adult *D. suzukii* individuals. The first captures of *D. suzukii* during the monitoring were between September and November, 2011 in a plum orchard, in the experimental field in Dossenheim (10 ♂, 1 ♀) and in a vineyard in Siebeldingen (3 ♂, 1 ♀). The JKI Dar captured the first individuals (12 ♂, 6 ♀) in August 2013 in a commercial cherry orchard near Nieder-Beerbach, Germany. 

**Figure 3:**
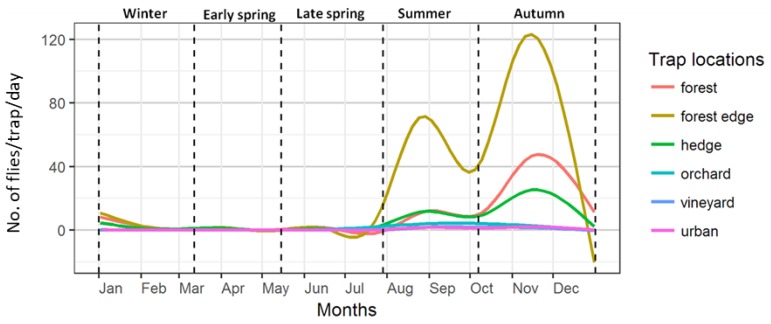
Smoothed LOESS curves of daily trap captures in different habitat types from 2011–2017. The vertical dotted lines mark the phenology phases (top; see Table 2 for a description).

**Figure 4:**
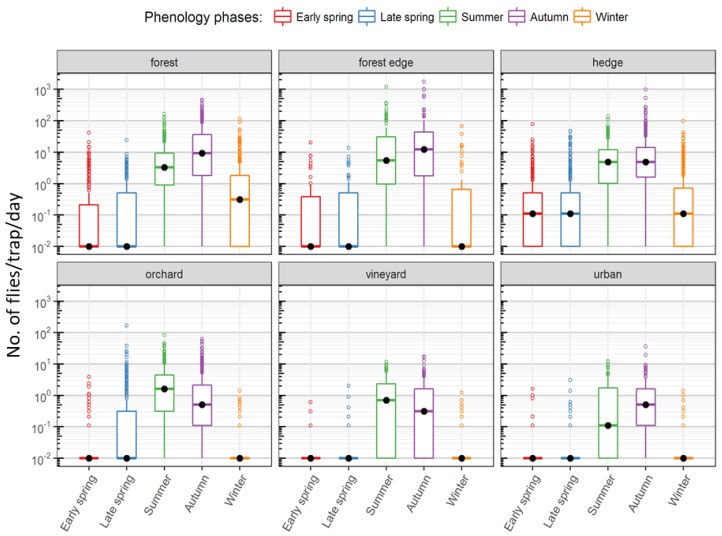
Box plots of daily captures in each sampled habitat type grouped by season. The median values represented by the bold black dots in each box plot highlight the variations in the seasonal dynamics of the captures in each sampled habitat type. The box represents the inter-quartile range (IQR) and the band inside the median. The whiskers represent data that are within 1.5 IQR below or above the first and the third quartiles, respectively. The outliers are represented by dots beyond the whiskers. Note: the Y-axis is log-scaled (captures/day/trap + 0.01).

**Figure 5:**
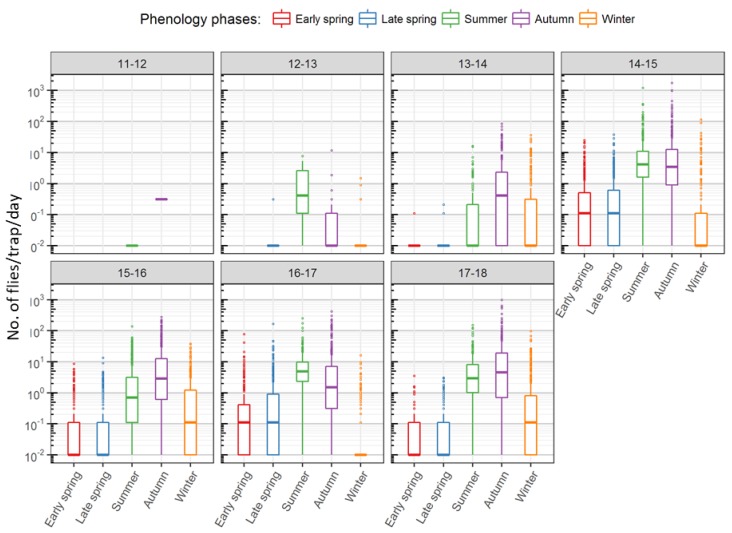
Box plots of captures/trap/day in each *D. suzukii*-year, grouped by SWD phenology. For a description of the box plots, see Figure 4. Note: the Y-axis is log-scaled (captures/day/trap + 0.01).

The mean annual trap activity followed population build-up starting in late spring and reaching a peak in summer. Forests and hedges recorded a second peak in autumn, after a brief decline in October (Figure 3). Capture rates steadily declined in winter to the very low numbers seen in early spring and the first half of late spring with a brief increase in April (Figure 3 and Figure S3). They also varied greatly between the sampled habitat types. Traps in forests, hedges and forest borders recorded the highest numbers, in peak seasons, over 1000 flies per day. In contrast, traps in fruit crops and vineyards recorded no more than a maximum of 50–100 flies per day (Figure 4). The plot of all trap captures together showed a distinct two-peak dynamic with higher captures in summer and autumn. Upon closer scrutiny, the two-peak dynamic is to be seen only in the case of forested areas and hedge traps and also not in all years, while those in vineyards and orchards show a decline already from the onset of cold weather (Figure 3). Though the highest number of captures in forested areas and hedges was in autumn, in some years, the median captures in summer were higher than in autumn (Figure 5). In most years, traps in forests and hedges captured consistently more flies in autumn than in summer, whereas traps in orchards captured more flies in summer than in autumn. Hedges and forests accounted for a much higher share of winter captures compared to other locations (Figure 4 and Figure S4).

**Figure 6:**
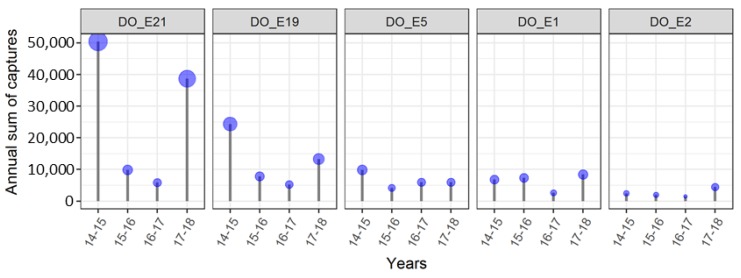
Yearly sum of captured individuals for five traps installed at/near the forest border. Traps DO_E21, DO_E19, DO_E1 and DO_E5 are located near the forest edge, while DO_E2 is located in the forest, 700 m from the forest border.

Five traps with very high capture rates were selected for a detailed analysis of the effects of land use on their capture rates (Figure 6 and Table S1). These traps were located on the borders of the Forest of Odes next to the Rhine valley with the highest captures in 2014–2015 and 2017–2018. Two traps (DO_E21 and DO_E19) were located prominently on a small mountain ledge, close to orchards/vineyards and the forest. Both traps captured between five- and 10-times more individuals over the years than trap DO_E5, which is also close to orchards/vineyards and shrubberies. The trap DO_E1 on the forest border, next to the urban area (Dossenheim) and a quarry captured less individuals than the above-mentioned traps, whereas the trap DO_E2, located deeper in the woods with only trees and no shrubberies nearby, and hence farther than the others from forest borders, orchards or urban areas, captured the lowest numbers.

Overall, an equal number of females and males were captured (50.9:49.1%), with small variations over the seasons. Slightly more females were captured in early spring, late spring and winter compared to summer or autumn (Table 5). The pattern was fairly similar across the different locations except in vineyards where more females than males were captured in summer.

**Figure 7:**
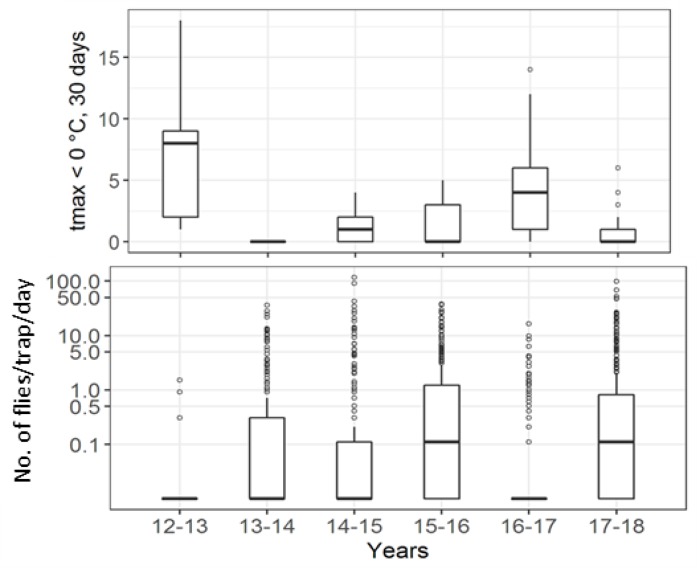
Winter captures: (top) the number of days when tmax < 0 °C in the 30 days before the trap content collection for each trap deployment, (bottom) captures/day from each trap deployment. Both panels are per *D. suzukii*-year. Note: log-scaled y-axis on the bottom panel.

Though *D. suzukii* was observed already in 2011, the next two years, 2012 and 2013, registered far less captures compared to the years after. Following the winter of 2013–2014, the warmest in the period with zero days below freezing, there was a comparable surge in the captures afterwards, never reaching values as low as those in 2012 and 2013. For studying the relationship between weather and captures, only capture data from 2013 onwards were used as *D. suzukii* appears to have successfully established in the monitored areas during the warm winter of 2013–2014. While the low temperatures in autumn were not accompanied by a corresponding change in capture rates, the higher number of ice days in the winter of 2016/2017 was reflected in the lowest winter captures from this year (Figure 7).

**Figure 8:**
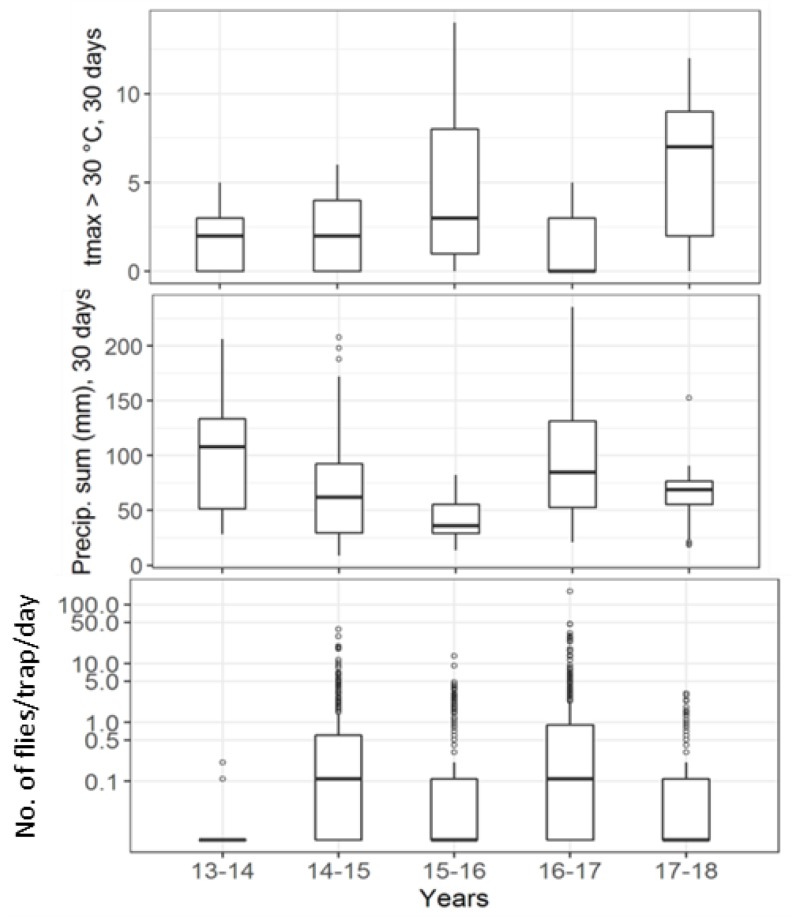
Late spring captures: (top) the number of days when tmax > 30 °C and (middle) precipitation sum (mm) in the 30 days before the traps were emptied for each trap deployment; (bottom) captures/day from each trap deployment. Both panels are per *D. suzukii*-year. Note: log-scaled y-axis on the bottom panel.

**Figure 9:**
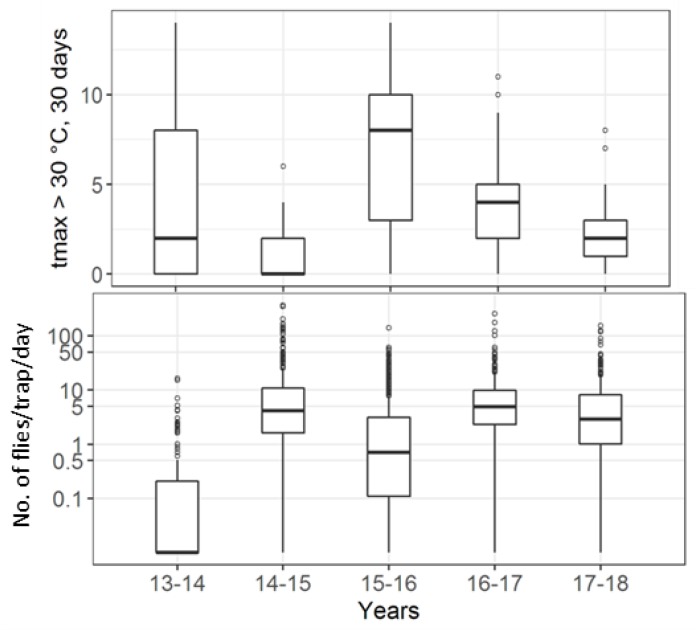
Summer captures: (top) the number of days when tmax > 30 °C in the 30 days before the traps were emptied for each trap deployment; (bottom) captures/day from each trap and trap deployment. Both panels are per *D. suzukii*-year. Note: log-scaled y-axis on the bottom panel.

During the monitoring period, the summer of 2017 was the hottest followed by that of 2015, resulting in the low late spring capture rates compared to those of 2014 and 2016. The 2014 and 2016 late springs also received more precipitation than those of 2015 or 2017 (Figure 8). The low summer captures in 2015 also corresponded to the high number of days when maximum temperature exceeded 30 °C this year (Figure 9).

The significant parameters from regressing seasonal captures against weather parameters are given in Table 6. Both late spring and summer captures were negatively associated with maximum temperatures above 30 °C. Temperatures in the range of 20 °C–30 °C defined as optimal weather for *D. suzukii* were positively associated with captures in late spring, but negatively associated for summer captures. Both frost and ice days were correlated with a decrease in capture rates in the cold seasons, while daily mean temperature (mean) and precipitation (sum) were positively correlated.

## 4. Discussion

In this study, we analyzed the monitoring data of the JKI in the upper Rhine valley in southwest Germany. The study started a few months before *D. suzukii* appeared for the first time in Germany, in August 2011, and followed its establishment in the succeeding years.

*Drosophila suzukii* has been present since at least late 2011 in southwest Germany. Currently, it is widespread and overwinters successfully in the upper Rhine valley. We suppose that the mild winter of 2013–2014 helped to establish a stable population in this part of Germany. The trap activity of this region was comparable to Northern Italy with a similar climate [27,28].

We split the typical annual population cycle of *D. suzukii* into five phenology phases (early spring, late spring, summer, autumn and winter) to better identify factors that affect the different phases of trap activity. Both weather conditions and habitat type influenced trap activity. The influence of habitat type on the trap captures is manifested in the regular, seasonal changes in the trap activity of *D. suzukii*.

The summer increase of trap captures and the following peak coincide with the ripening of most wild and cultivated host fruits of *D. suzukii* [11,41]. The single annual peak in summer in orchard and vineyard traps suggests that those sites are not suitable overwintering habitats and *D. suzukii* has to re-immigrate each year, whereas forested areas appear to be the favored habitat year-round. The increase of trap captures from late spring onwards until the autumn peak in forests, forest borders and hedges has also been noted in other temperate regions [20,22,24,42]. The single peak in late spring/summer in fruit crops and vineyards is quite similar to the findings in northwestern Italy [28]. We suppose that when attractive food sources are present, the traps are less attractive to the flies, such as in orchards, but in forests, the same baits are more attractive to the flies due to less competition between ACV-baited traps and attractive food sources. Further, forests may provide better habitat conditions when it is too hot in summer or too cold in winter. The presence of berries in hedges or in the forested areas [11,36,43] at various stages of ripeness partly all throughout the year could be an additional factor why forested areas and hedges always recorded very high capture rates and microorganisms growing on the surface of evergreen trees in the cold bottleneck seasons may also serve as food resources, supporting *D. suzukii* populations [13]. Thus, we cannot conclude whether higher trap captures in forests result from a higher *D. suzukii* abundance or a higher attractivity of traps. The winter decline and low captures in early spring have also been noted in California [22,44], northern Italy [28,45] and Canada [20].

The five traps at the forest border of the Forest of Odes suggest a distinct importance of forest borders to *D. suzukii*. Traps with the highest captures, located prominently on a small mountain ledge, were especially attractive in autumn, when most trees in orchards and vineyards have already lost their leaves. We assume that *D. suzukii* migrates from orchards/vineyards in the plains to forested areas in autumn in search of nutrition and overwintering habitats. DO_E21, in particular, offers such habitats since it is located on a *Robinia pseudoacacia* overgrown with *Hedera helix*. Our findings are in agreement with Tait et al. (2018) [46] that *D. suzukii* migrates between different habitats, depending on the season, searching for suitable overwintering habitats. Since DO_E2 captured less individuals than DO_E1, we assume that *D. suzukii* migrates into sheltered overwintering sites such as hedges and forested areas as long as it can find shrubbery. The attractiveness of the traps appears to be strongly influenced by the immediate habitat and the seasonal changes in the habitat with respect to the availability of nourishment and shelter. We recommend taking this into consideration when estimating in-field fly density based on trap captures or when trap capture data are treated as representative of fly density.

Based on our seven-year monitoring data with much higher capture rates in forests than in fruit orchards or vineyards even in the fruiting season, we question the reliability of the estimation of the actual density of *D. suzukii* in the field using ACV traps. When natural food sources are available, the flies seem to prefer the ripe fruits markedly more than the ACV mixture, resulting in an under-representation of the fly density in the fruit orchards and vineyards during the fruiting season. For economic reasons and to have comparable data over the monitoring period, we did not change our trap designs during the study. Our trap designs may have resulted in relatively conservative estimates of the abundance compared to other traps and/or baits that are now available [47,48,49]. However, it was already suggested that every individual fly captured in an ACV-baited trap represents 198 flies in a 2.7-ha cherry orchard [50]. These findings strengthen the argument that trap captures should be interpreted with caution [5,29,44,51].

We tested the capture data against temperature thresholds that were found to affect *D. suzukii* individuals and populations significantly in field and laboratory studies. The decline in trap captures when tmax > 30 °C in late spring was noted previously [22,30]. Further, it was already suggested that in lab assays, temperatures above 30 °C reduce reproduction or even inhibit it [52,53]. Although our weather data were of quite high resolution (1 × 1 km^2^), it would be impossible to simulate the authentic field conditions that could vary in much smaller units that provide suitable shelters for *D. suzukii*. We also found that the years with high late spring captures correlated with high precipitation. Whether this is due to the effect of precipitation on *D. suzukii* host plants or the higher relative humidity favoring *D. suzukii* was beyond the scope of our study.

Since the climate in southwestern Germany is more akin to that of Northern Italy [28] than to California [22] or Canada [20], we expected *D. suzukii* population dynamics to be similar to that observed in northern Italy. The winter of 2013/2014, with zero days below freezing and only a few cold days, was the mildest during the whole monitoring period. Survival success during this mild winter could have played a key role in the excessive damages observed in the following spring in this region. Next, low temperatures in autumn do not seem to be correlated with low capture rates, whereas ice-days in winter are followed by a decrease in trap captures over a longer period (e.g., winter of 2016/2017). In contrast to previously published findings [20], we did not find any conclusive relationships between mild winter days and earlier fly appearance in the traps the following spring.

Both females and males were captured all throughout the year, but the number of males was slightly higher during the peak seasons (summer and autumn), and previously published studies showed less attractiveness of ACV-baited traps to female *D. suzukii* the more mature eggs their ovaries contained [1,54]. However, we assume that a combination of this and a higher dispersal of males to ensure gene flow results in higher capture rates of males during the peak seasons. More females were captured in the colder seasons when trap captures were lower, suggesting the better adaptability of female winter morphs to colder periods with insufficient food sources. The higher cold tolerance of females was observed in many other studies, as well [20,21,42,55,56]. However, reports are not conclusive. Ryan et al. [52] did not find any difference between sexes in their response to low temperatures, while Enriquez and Colinet [57] even suggested that males are better adapted to survive through winters. 

## 5. Conclusions

Through our monitoring program we were able to record the first appearance of *D. suzukii* in Germany in 2011 and follow its establishment over the following years. Despite its first occurrence in 2011, it was the mild winter of 2013/2014 that helped *D. suzukii* the firm establishment in southwest Germany. The successful establishment in this region is proof of its adaptability to cold winters, hot summers, and fluctuating temperatures, in a small structured landscape. 

Further, our analysis confirms that temperature thresholds for in-field development and survival should be re-adjusted with respect to further meteorological (e.g., global radiation) and habitat parameters as conclusions based on laboratory experiments cannot be easily juxtaposed with those based on field studies. While laboratory studies are carried out in a controlled environment, numerous factors are at play in field studies. Next, trap captures do not reflect actual in-field fly densities and we still lack information about realistic in-field population densities since the attractiveness of ACV baited traps varies significantly between habitats and seasons.

A detailed analysis of the effect of landscape on *D. suzukii* captures and determination of weather conditions was beyond the scope of the study. However, it must be noted that the traps were spatially quite clustered and did not map large variations in weather. An analysis of a country-wide monitoring is necessary to validate the results on a bigger scale.

## Figures and Tables

**Table 1:** Total number of traps per JKI site in each habitat type and the marginal sums.

Institute	Forest	Forest Border	Hedge	Orchards	Vineyard	Urban Area	Total
JKI Dos	33	2	18	15	0	0	68
JKI Sie	1	1	5	6	4	2	19
JKI Dar	3	1	3	4	0	2	13
Total No. of traps	37	4	26	25	4	4	100

**Table 2:** Definitions of the five phenology classes representing the activity patterns of adult *D. suzukii*.

Seasonal Classes	Start-End	Day of Year	Observation
Early spring	March 12–May 15	71–135	Lowest capture rate
Late spring	May 16–July 29	136–210	Increase in trap captures, emergence of summer morph
Summer	July 30–October 7	211–280	First peak in number of captures
Autumn	October 8–December 31	281–366	Second peak in number of captures, emergence of winter morph
Winter	January 1–March 11	1–70	Winter decline of captures

**Table 3:** Weather indices created to test the relationship between weather and capture rates of *D. suzukii*. All parameters were calculated for the 30-day period before the trap contents were collected. Expected relationships are coded ‘−’ as unfavorable and ‘+’ as favorable to *D. suzukii*.

Weather Indices	Description	Expected Effect on *D. suzukii*
T_max_ < 0 °C	Ice days	−
T_min_ < 0 °C	Frost days	−
T_max_ ≤ 8 °C	Cold days	−
20 °C ≤ T_mean_ ≤ 30 °C	Optimal development range	+
T_max_ > 30 °C	Hot days	−
T_mean_ °C	Average temperature	Varying with season

**Table 4:** The number of traps monitored by each institute for each *D. suzukii*-year. Given in brackets are the number of traps that were not included in this study, as these traps were in operation before the first catch of *D. suzukii*.

Operator	2011–2012	2012–2013	2013–2014	2014–2015	2015–2016	2016–2017	2017–2018
**JKI Dar**	(0) 0	(8) 0	(10) 3	10	13	13	6
**JKI Dos**	(5) 1	11	23	60	56	61	45
**JKI Sie**	(19) 0	16	17	17	19	19	17
**Total**	(24) 1	27	43	87	88	93	68

**Table 5:** Percentages of male and female *D. suzukii* captures in different seasons over the monitoring period.

*D. suzukii* Phenology	Males%	Females%
Early spring	39.9	60.1
Late spring	44.5	55.5
Summer	52	48
Autumn	55.1	44.9
Winter	40.3	59.7

**Table 6:** Results from regression tests of seasonal captures against weather parameters. Shown are only parameters with significant *p*-values. ‘coeff’ is the regression coefficient, ‘se’ the standard error.

Season	Weather Parameter	Coeff	se	*p*-Value
Late spring	20 °C ≤ Tmean ≤ 30 °C (days)	0.134	0.008	<0.001
Late spring	Mean of tmean (°C)	0.309	0.020	<0.001
Summer	20 °C ≤ Tmean ≤ 30 °C (days)	−0.045	0.005	<0.001
Summer	Mean of tmean (°C)	−0.163	0.014	<0.001
Summer	tmax > 30 °C (days)	−0.131	0.009	<0.001
Late spring and autumn	tmax > 30 °C (days)	−0.102	0.009	<0.001
Autumn, winter and early spring	tmin < 0 °C (days)	−0.092	0.005	<0.001
Autumn, winter and early spring	Precipitation sum (mm)	0.014	0.001	<0.001
Autumn, winter and early spring	Mean of tmean (°C)	0.110	0.009	<0.001
Autumn, winter and early spring	tmax < 0 °C (days)	−0.350	0.026	<0.001
Autumn, winter and early spring	tmean ≤ 8 °C (days)	−0.026	0.003	<0.001

## Supplementary Materials

The following are available online at http://www.mdpi.com/2075-4450/9/4/125/s1: Figure S1. (a) Example of a new cup, (b) the used stencil for standardized trap construction and (c) the prepared trap. Figure S2. Partial counting of large captures (>5 mL): (a) petri dish (Ø 6 cm) split into six equal sections for catches <15 mL; (b) petri dish (Ø 14 cm) split into eight equal sections for catches >15 mL. The sections were made with wires attached to a plastic ring insert placed inside the petri dish. Figure S3. Captures per day from each trap over the monitoring period (2011–2018) with a smoothing curve (blue), based on which the five seasonal categories were created. The bubble size (small to big) is proportional to the capture size. Note: the y-axis is log-scaled. Figure S4. Box plots of captures/trap/day in each *D. suzukii*-year, grouped by sampled habitat types and *D. suzukii* phenology. The box represents the inter-quartile range (IQR) and the band inside the median. The whiskers represent data that are within 1.5 IQR below or above the first and the third quartiles. The outliers are represented by dots beyond the whiskers. Note: the Y-axis is log-scaled (captures/day/trap + 0.01). Figure S5. Annual sum of *D. suzukii* captures in the *D. suzukii*-years from 2014–2015 until 2017–2018 grouped by *D. suzukii* phenology and site/trap. The size of the blue bubble is proportional to the annual sum. Note: the y-axis scale varies between rows. Table S1. Distances of select traps from the forest border, orchards/vineyards and urban areas, coordinates and altitude. The five traps shown here are located in and around the Forest of Odes and captured significantly higher individuals than the rest of the traps from this study.
