# Supplementary material for: Explorative Data Analysis of Drosophila suzukii Trap Catches from a Seven-Year Monitoring Program in Southwest Germany
Source: Insects. 2018 Sep 24;9(4):125. doi: 10.3390/insects9040125 (PMC6315382; doi:10.3390/insects9040125)
Supplement: Supplementary file 1 [file insects-09-00125-s001.pdf]

insects

MDPI

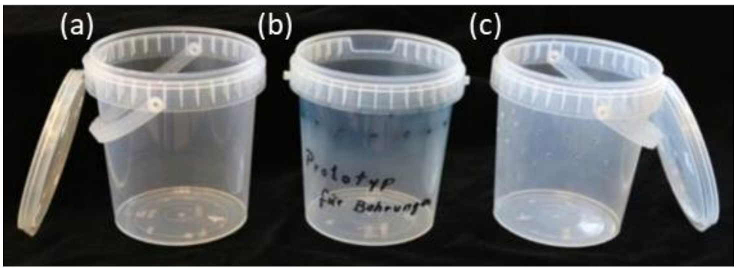

Figure S1. (a) Example of a new cup, (b) the used stencil for standardized trap construction and (c) the prepared trap.

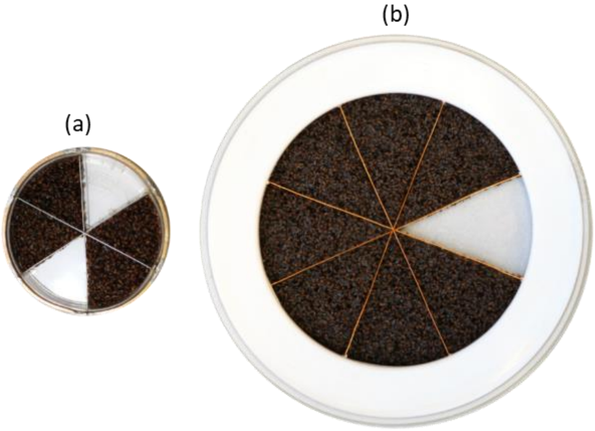

Figure S2. Partial counting of large captures (> 5 ml): (a) petri dish (Ø 6 cm) split into six equal sections for catches < 15 ml; (b) petri dish (Ø 14 cm) split into eight equal sections for catches >15 ml. The sections were made with wires attached to a plastic ring insert placed inside the petri dish.

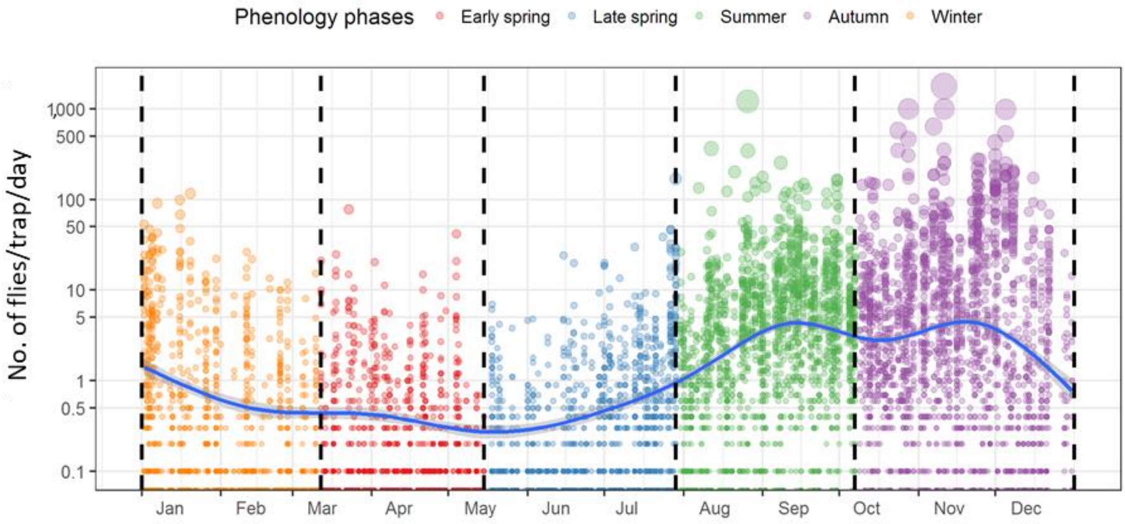

Figure S3. Captures per day from each trap over the monitoring period (2011 – 2018) with a smoothing curve (blue), based on which the five seasonal categories were created. The bubble size (small to big) is proportional to the capture size. Note: the y-axis is log-scaled.

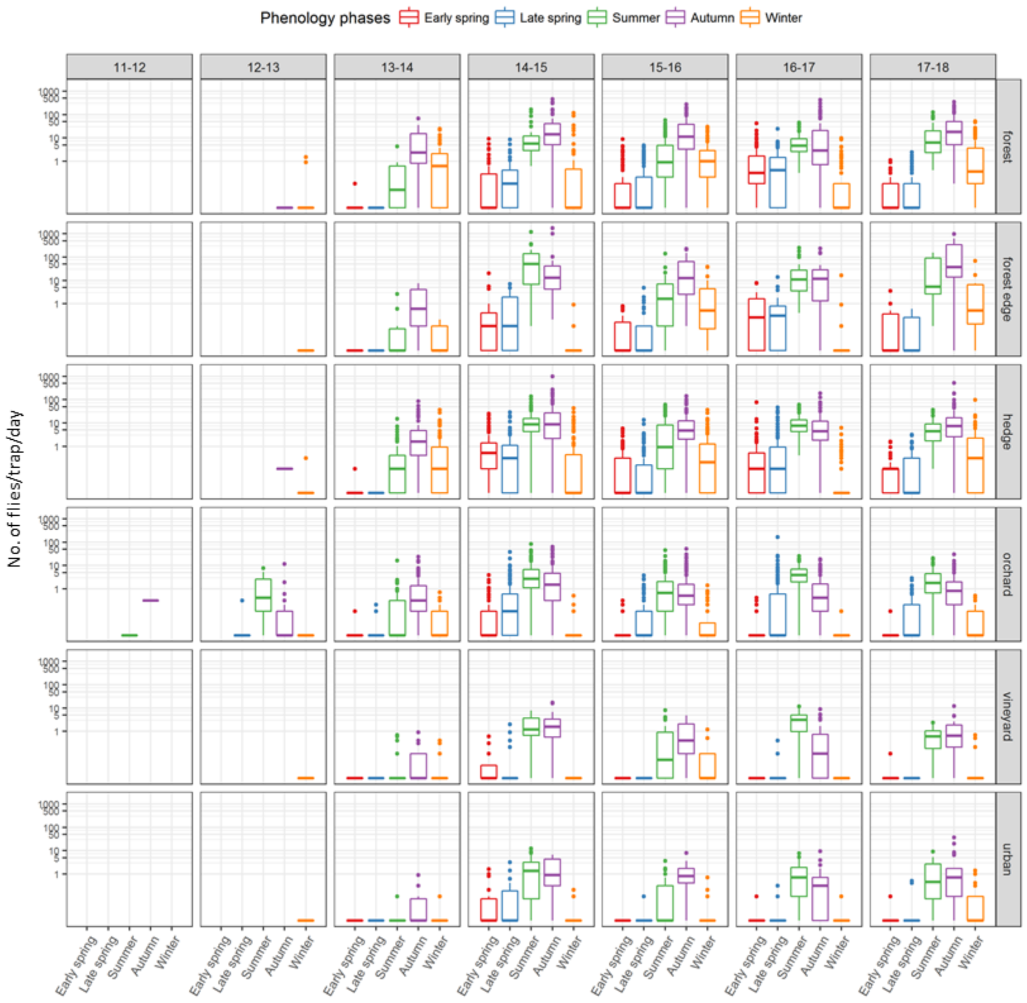

Figure S4. Box plots of captures/trap/day in each *D. suzukii* - year, grouped by sampled habitat types and *D. suzukii* phenology. The box represents the inter-quartile range (IQR) and the band inside, the median. The whiskers represent data that are within 1.5 IQR below or above the 1st and the 3rd quartiles. The outliers are represented by dots beyond the whiskers. Note.: Y-axis is log-scaled (captures/day/trap + 0.01).

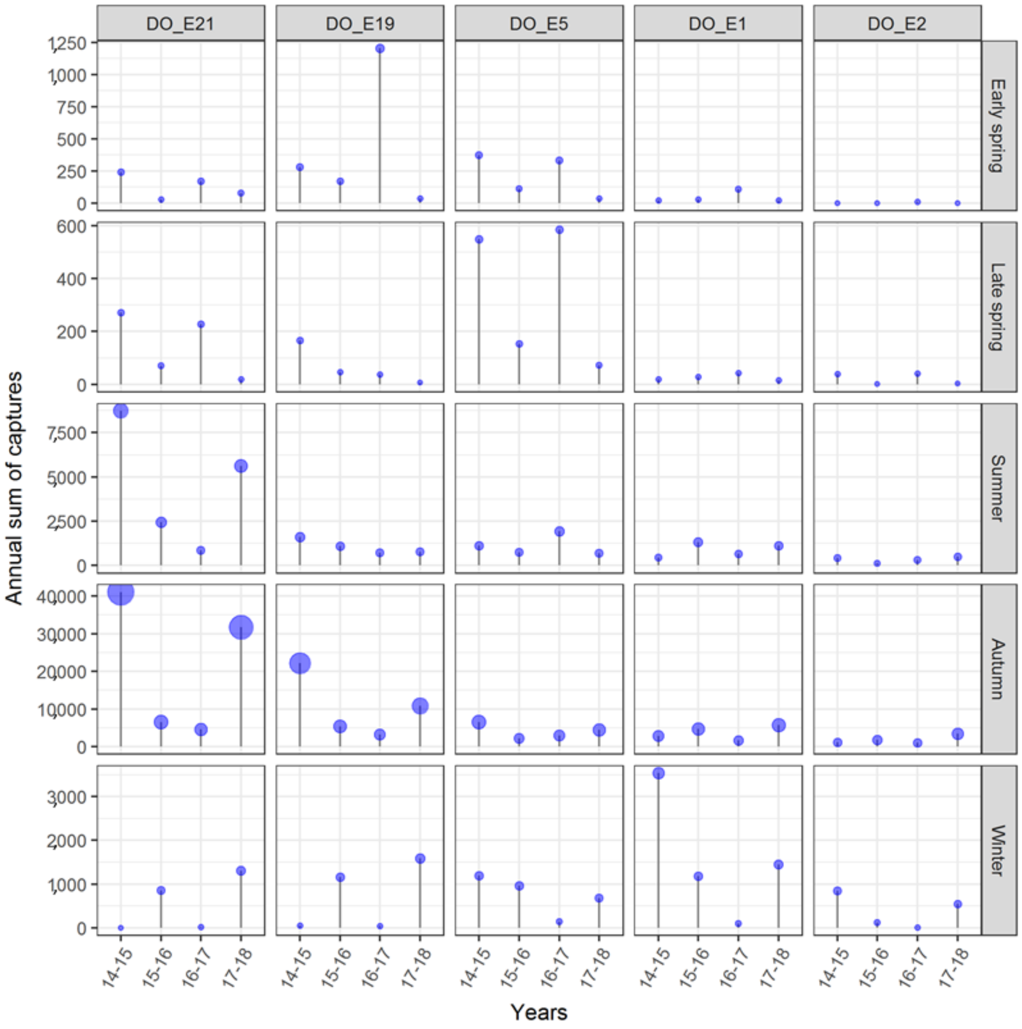

Figure S5. Annual sum of *D. suzukii* captures in the *D. suzukii* -years from 2014-2015 until 2017-2018 grouped by *D. suzukii* -phenology and site/trap. The size of the blue bubble is proportional to the annual sum; Note: y-axis scale varies between rows.

Table S1. Distances of select traps from the forest border, orchards/vineyards and urban areas, coordinates and altitude. The five traps shown here are located in and around the Forest of Odes and captured significantly higher individuals than the rest of the traps from this study.

Trap name	Trap location	Distance to forest border (m)	Distance to orchard/vineyard (m)	Distance to residential area (m)	Coordinates	Altitude asl (m)
DO_E1	On the forest border	0	300	110	E8.68300703 N49.44914396	236
DO_E2	In the forest	700	900	165	E8.69164199 N49.45003001	285
DO_E5	Near the forest border	150	1	390	E8.67215700 N49.46331901	167
DO_E19	On the forest border	17	5	170	E8.67126801 N9.47380100	237
DO_E21	On the forest border	13	10	100	E 8.67096903 N49.47397602	231
